# Inhalatory sedation in postoperative neurovascular surgery patients

**DOI:** 10.1186/2197-425X-3-S1-A324

**Published:** 2015-10-01

**Authors:** R Badenes, A Belltall, V Chisbert, M De Fez, A Lozano, P Valls, I Fuentes, E Gracia, F Bilotta

**Affiliations:** Hospital Clinico Universitario de Valencia, Anesthesiology and Surgical-Trauma Intensive Care, Valencia, Spain; Hospital Clinico Universitario de Valencia, Valencia, Spain; Hospital de la Ribera, Alzira, Spain; Sapienza University of Rome, Rome, Italy

## Introduction

Sedation is used almost universally in the care of Intensive Care Unit (ICU). The action and side-effects of intravenous drugs in the severely ill patient population of an ICU are difficult to control. The Anesthetic-Conserving Device (AnaConDa) can be used to administer inhaled anesthetics using an ICU ventilator. Inhaled sedation is efficient and easily controllable; in low concentrations it causes minimal changes in the patient and very little interference with hemodynamics. Awakening after inhaled sedation is quick and predictable.

## Objectives

The main goal is to assess the safety and efficacy of using the AnaConDa with sevoflurane when maintaining sedation after neurovascular surgery.

## Methods

Prospective observational study of 16 consecutive patients in the Surgical Intensive Care Unit (SICU) of a tertiary university hospital after neurovascular between July 2014 and December 2014. We studied 16 SICU patients who received sevoflurane via the AnaConDa. The patients were under sevoflurane sedation administered with the AnaConDa placed in the inhalation tube. The sevoflurane dose was set using the Belda et al. [1] normogram to give an end-tidal concentration of sevoflurane between 0.5 and 0.7% on the basis of data from a gas analyzer. Fast-track extubation protocol was used.

## Results

The sedation goal (Richmond -4,-5) was reached with sevoflurane in all 16 patients. The mean (SD) time each patient were under sedation with the AnaConDa in place was 72 (11.44) min. The end-tidal concentration of sevoflurane never exceeded 1.1%. Richmond agitation-sedation scale were -5 at 60 min in all cases; Deeper sedation was desired for the first 60 min to avoid awakening related to relaxing. The mean time until awakening was 3.12 min (range 1-11 min). The mean time until extubation was 8 (6.44) min. Hemodynamic changes were nonsignificant, and no renal or hepatic dysfunctions were observed.

## Conclusions

Routine SICU postoperative neurovascular surgery patients sevoflurane sedation with the AnaConDa is easily feasible, effective, safe, and has a relatively short awakening period. With this device, it is possible to monitor the concentration administered. The use of volatile anesthetics on the ICU could adopt a permanent position in various intensive care sedation concepts in future. It may be possible thereby to optimize the treatment process both in medical and economical terms. It is the first study using sevoflurane sedation in postoperative neurovascular surgery patients.
